# Decision support for assessment of left ventricular diastolic function

**DOI:** 10.14814/phy2.13815

**Published:** 2018-08-20

**Authors:** Éva Tamás, Eva Nylander

**Affiliations:** ^1^ Department of Cardiothoracic and Vascular Surgery Department of Medical and Health Sciences Linköping University Linköping Sweden; ^2^ Department of Clinical Physiology Department of Medical and Health Sciences Linköping University Linköping Sweden

**Keywords:** Diastolic function, echocardiography, left ventricular function

## Abstract

Echocardiographic assessment of the left ventricular diastolic function (LVDF), an integrated part of evaluation of left ventricular function is still a delicate task and is performed with substantial inter‐rater variability. Therefore, we aimed to create and evaluate a guidelines‐based automated decision support. An algorithm was created for a hierarchical analysis of LVDF based on variables as recommended by the latest guidelines. Age‐adjusted normal ranges were pooled from previously published studies into an integrated reference table. For proof‐of‐concept, 20 echocardiographic examinations were analyzed offline by four experienced physicians with more than 10 years of echocardiographic experience. The first assessments were to be performed as they would be in the clinical practice. Six months later, the assessments were repeated based on the 2017 ASE/EACVI guidelines. The overall inter‐rater agreement for the first clinical assessments was moderate, while the guidelines‐based assessments had only fair inter‐rater agreement. Both kinds of manual assessment had poor agreement with the standardized automated assessment algorithm of LVDF. In conclusion, the presented automated decision support for evaluation of diastolic LV function by Doppler echocardiography is mainly based on current guidelines involving multiple parameters in combination. Incorporating age dependency aspects in our program (available for use at https://liu.se/en/research/left-ventricular-diastolic-function-decision-support) enhances the accuracy of the evaluation and reduces variability in evaluation of LVDF. The large inter‐rater variation in classification in this study also underscores the usefulness of tools to support a standardized evaluation.

## Introduction

Echocardiographic assessment of the left ventricular diastolic function (LVDF) has become an integrated part of clinical routine. The American Society of Echocardiography (ASE) and the European Association of Cardiovascular Imaging (EACVI) have provided clinicians with guidelines and recommendations to facilitate standardized assessment (Nagueh et al. 2016). However, evaluation of LVDF is still a delicate task and it is performed with substantial inter‐rater variability. (Unzek et al. 2011; Selmeryd et al. 2016, 2018). A possible explanation for this variability is that classification of diastolic function relies on a combination of variables, of which the relative importance has varied over time and with generations of methodologies and guidelines. Each variable is also subject to measurement errors that may be multiplied when combining several parameters, and different cutoffs or reference values have also been used for classification. How to assess in case of discordant information from the different diastolic function variables may also vary and influence classification. The great complexity of echocardiographic measures used for the assessment of the LVDF and the age dependency of variables leaves a wide gap for interpretation. Furthermore, the flowcharts in the latest guidelines do not include age‐related reference values, though it is recommended to take age into consideration when evaluating LVDF. Several studies have assessed normal values of LVDF (Gentile et al. 1997; Munagala et al. 2003; Dalen et al. 2010; Caballero et al. 2015; Hagstrom et al. 2017) in relatively large healthy populations but age categories vary in these studies, which makes cross‐referencing complicated.

Our aim was to create (1) a guidelines‐based automated decision support algorithm, and (2) to evaluate and compare inter‐rater agreement for manual LVDF assessments and the decision support algorithm.

## Material and Method

**Figure 1:**
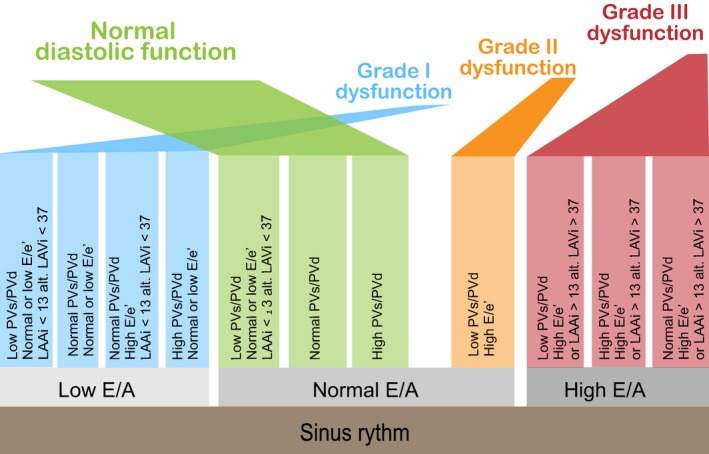
Assessment algorithm for standardized automated evaluation of LVDF. The assessment is based on E/A, PVs/PVd, E/e’, LAAi or LAVi. The algorithm first compares these diastolic parameters to age‐corrected normal values, and values are sorted into the categories “low”, “normal”, and “high”. Thereafter, the categorized variables are entered into the analysis in hierarchical order based on the latest recommendations from guidelines for evaluation of LVDF, and the LVDF category is presented automatically. In the case of conflicting values or missing key variables, a request for manual assessment is presented. LVDF, left ventricular diastolic function.

An automated standardized analysis algorithm (Fig. 1) was created (Visual Basic for Applications for Excel 2016, Microsoft Inc.) for a hierarchical analysis of LVDF based on guidelines’ recommendations (Nagueh et al. 2016). Normal ranges were pooled and adjusted from previously published studies (Gentile et al. 1997; Munagala et al. 2003; Caballero et al. 2015) in an integrated reference table (Table 1). For age‐related reference values, we mainly used the data published by Munagala et al. (2003), due to its population‐based cohort, and the inclusion of more elderly patients, with data displayed more selectively for higher age groups than the materials published by, for example, Caballero et al. (2015). However, to cover younger patient groups, we added information on patients below 45 years of age from Caballero et al. (2015) and on pulmonary venous systolic and diastolic velocities provided by Gentile et al. (1997). This resulted in a reference table with pooled and age‐adjusted data from these sources (Table 1). The ranges were derived from normal reference values using one standard deviation in order to reduce the risk of false negative diagnoses.

**Table 1:** Age‐related reference values for diastolic parameters

	<45 years		45–49 years		50–54 years		55–59 years		60–64 years		65–69 years		>70 years	
	Min	Max	Min	Max	Min	Max	Min	Max	Min	Max	Min	Max	Min	Max
E (m/sec)	0.66^b^	0.98^b^	0.50	0.90	0.50	0.90	0.50	0.90	0.50	0.90	0.40	0.80	0.40	1.00
A (m/sec)	0.37^b^	0.63^b^	0.30	0.70	0.40	0.80	0.40	0.90	0.40	0.90	0.40	1.00	0.50	1.10
E/A	1.19^b^	2.23^b^	1.00	2.00	0.80	2.00	0.70	1.80	0.70	1.60	0.60	1.50	0.60	1.30
PV_s_ (m/sec)	0.34^c^	0.54^c^	0.40	0.80	0.40	0.80	0.40	0.80	0.40	0.80	0.50	0.80	0.40	0.80
PV_d_ (m/sec)	0.47^c^	0.64^c^	0.30	0.60	0.30	0.60	0.30	0.60	0.30	0.60	0.30	0.60	0.30	0.60
PV_s_/PV_d_	0.56^c^	1.07^c^	0.86	2.00	1.00	2.00	1.00	2.00	1.00	2.25	1.00	2.50	1.00	2.50
e’ S (cm/sec)	7.00^a^	14.00^a^	7.00	14.00	6.00	14.00	5.00	12.00	6.00	13.00	5.00	11.00	5.00	11.00
a’ S (cm/sec)	7.00^a^	14.00^a^	7.00	14.00	8.00	14.00	8.00	15.00	9.00	15.00	9.00	15.00	9.00	15.00
E/e’ S	4.62^a^	11.25^a^	4.62	11.25	4.55	11.67	4.62	13.33	5.00	12.00	5.45	13.33	4.55	16.67
e’ L (cm/sec)	9.00^a^	17.00^a^	9.00	17.00	8.00	16.00	7.00	15.00	7.00	15.00	7.00	12.00	5.00	11.00
a’ L (cm/sec)	7.00^a^	16.00^a^	7.00	16.00	7.00	15.00	8.00	16.00	8.00	17.00	9.00	16.00	8.00	18.00
E/e’ L	3.75^a^	7.78^a^	3.75	7.78	3.75	8.89	3.85	10.00	4.62	8.89	4.17	11.25	5.00	14.00
E/e’ mean	4.19^a^	9.52^a^	4.19	9.52	4.15	10.28	4.24	11.67	4.81	10.45	4.81	12.29	4.78	15.34

Collated age‐related reference values (min‐max ± 1 SD) for diastolic parameters are presented. (a) extrapolated values based on normal values by Munagala et al. (2003), (b) Caballero et al. (2015) and (c) Gentile et al. (1997) E: early diastolic mitral flow velocity.

i, Indexed for body surface area; E (m/sec), Early diastolic mitral flow velocity; A (m/sec), Late diastolic mitral flow velocity; PVs (m/sec), Pulmonary vein systolic flow velocity; PVd (m/sec), Pulmonary vein diastolic flow velocity; e’ S (cm/sec), Early diastolic septal myocardial velocity; a’ S (cm/sec), Late diastolic septal myocardial velocity; e’ L (cm/sec), Early diastolic lateral myocardial velocity; a’ L (cm/sec), Late diastolic lateral myocardial velocity; E/A, Ratio of early‐ to late diastolic mitral flow velocity; E/e, Ratio of early diastolic mitral flow velocity to early diastolic myocardial velocity.

Current guidelines (Nagueh et al. 2016) propose different algorithms for evaluation of patients with normal and reduced LVEF, respectively. As persons with “myocardial disease and normal LVEF” as well as patients with valve disease are also suggested to be handled by the latter algorithm and our main interest was in classifying diastolic function in patients with heart disease, we set out to adapt this algorithm for the standardized evaluation support.

The guidelines‐suggested algorithm proposes a primary classification based on E/A ratio. In the text of the guideline document it is stated that age should be taken into consideration; however, this is not applied in the algorithm.

Therefore, we chose to classify patients′ diastolic function based primarily on E/A, as in the guidelines, but instead of using fixed cutoff values for E/A we defined the three classification groups as E/A lower than age‐related reference, higher than reference and within normal reference limits for age.

To support the classification, we added E/e′ (e′ averaged for septal and lateral basal LV), and pulmonary vein systolic/diastolic (PVs/PVd) velocity, both age‐related, and left atrial area indexed for BSA (LAAi) as described in Figure 1. In the case of contradictory information or missing data, a need for manual classification was signaled by our algorithm. Classification was then performed manually, according to the reference data as in the algorithm but with the addition of information from E‐deceleration time (age‐related) and tricuspid regurgitation velocity (>2.8 m/sec in concordance with recommendations).

For left atrial (LA) size, recommendations advocate biplane volume determination (Lang et al. 2005). Our original study design included a 4‐chamber view for LA size measurement but not 2‐chamber images with focus on the LA. Since single‐plane volume determinations cannot theoretically be superior to the area determination that they originate from, we have here used the 4‐chamber LA area (LAA) indexed for BSA. It has also been shown that the LAA was nearly equivalent to the left atrial volume index (LAVi) for the detection of moderate to severe diastolic dysfunction (grade II‐III) and that the specificity to predict a normal LA size of LAA compared to LAVi was 98% (Stefano et al. 2012). In our suggested decision support, the LA area may, however, be replaced by LA volume above or within reference limits, without altering the logic of the algorithm.

A batch of 20 echocardiographic examinations was prepared. The patients were randomly chosen (RANDOM.org) from a cohort of 397 patients with severe aortic stenosis who had been referred for operation during a 5‐year period at the Department of Cardiothoracic Surgery, University Hospital, Linköping, and who had been included in a study of preoperative characteristics and outcome. The sample represented a spectrum of diastolic function abnormalities and an age distribution representative of patients where diastolic function is often evaluated.

Echocardiographic images (Vivid E9 ultrasound system, GE Vingmed Ultrasound, Horten, Norway) were saved for offline analysis. LVDF was evaluated by four experienced physicians, clinical physiologists with more than 10 years of echocardiographic experience. These physicians were instructed to perform a first assessment, independently from each other, conducting the evaluation as they would do in their clinical practice. The assessments were repeated 6 months later for the same batch by the same clinical physiologists. This time they were instructed to perform the assessments based on the ASE/EACVI guidelines (Nagueh et al. 2016).

Diastolic dysfunction was categorized as grades I‐III. Measurements not fulfilling these criteria, and thus within normal range were denoted as “normal.”

Manual and automated assessments were compared by attribute assessment analysis and Fleiss’ kappa (*κ*) was computed to evaluate agreement among evaluations. Agreement was considered to be poor for *κ *< 0.20, fair for *κ *= 0.21–0.39, moderate for *κ *= 0.4–0.59, good for *κ *= 0.60–0.79 and very good for *κ *> 0.8 (Viera and Garrett 2005). It was anticipated that rater agreement would be 80% with a relative error of 30%; thus, a minimum of 17 cases was required for review to achieve 90% power (Bujang 2017). Significance was set as *P* < 0.05 (STATISTICA 13.1, Dell Inc., 2300 East 14th Street, Tulsa, OK 74104).

The study was approved by the Regional Ethical Review Board of Linköping (Ref. No. M198‐07) and every patient signed the informed consent for the study.

## Results and Discussion

The evaluated 20 patients were 72 ± 12 years old with a BSA 1.9 ± 0.2 m^2^ and BMI 24.6 ± 3.3 kg/m^2^ (mean ± SD). The male‐female ratio was 10:10.

**Figure 2:**
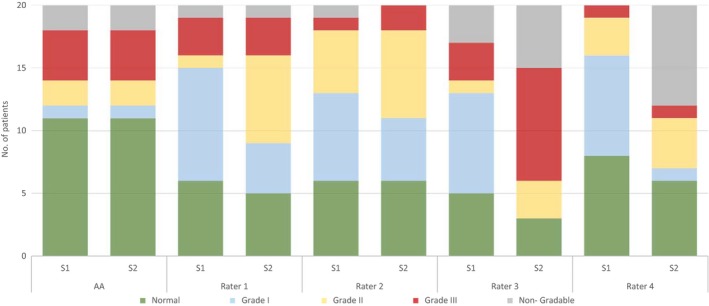
Left ventricular diastolic function by manual and standardized automated assessments. Number of patients in each left ventricular diastolic function category by standardized automated assessment (AA) and manual assessment according to clinical practice and guidelines‐based assessment (R: rater, S: series).

The overall inter‐rater agreement for the first clinical assessments was moderate while the guidelines‐based assessments 6 months later had only fair inter‐rater agreement. Both kinds of manual assessment had poor agreement with the standardized automated assessment algorithm of LVDF (Table 2). The only good inter‐rater agreement was found for normal LVDF assessed based on guidelines. Compared to manual rating by experienced observers, the automated assessment resulted in more patients tending to be classified within normal limits (Fig. 2).

**Table 2:** Inter‐rater agreement for manual and automated assessments of Left ventricular diastolic function

	Normal	Grade I	Grade II	Grade III	Non‐gradable	Overall
Inter‐rater agreement for clinical assessment	0.44	0.58	0.31	0.54	0.36	0.47
Inter‐rater agreement for guidelines‐based assessment	0.73	0.09	0.16	0.15	−0.1	0.24
Standardized assessment versus clinical assessment	0.35	−0.003	−0.1	0.14	−0.09	0.12
Standardized versus guidelines‐based assessment	0.35	−0.1	−0.1	0.2	0.003	0.13

Fleiss’ kappa is presented for the assessment agreement between different evaluations.

Evaluation of the LVDF is complex due not only to the number of variables to be considered but also the factors affecting these variables. For example, filling velocities through the mitral valve can be affected by concomitant aortic regurgitation. Also, because of annular calcification, mitral annulus motion affects the echocardiographic measurements and the variables are age‐dependent. We found poor inter‐rater agreement for assessment of the LVDF, in concordance with previous studies. Unzek et al. (2011) found that the ASE/EACVI 2009 guidelines helped to differentiate the group of patients with low filling pressure from patients with high filling pressure. However, the differentiation between subcategories showed poor agreement. When assessment of diastolic function was dichotomized for emergency medicine, defining diastolic dysfunction as septal e’ was <8 cm/s and if the lateral e’ was <10 cm/s, the inter‐rater agreement was found to be very good (Saul et al. 2016). Hence the discrepancy in the inter‐rater variability was reduced due to the simplified assessment algorithm. However, the correctness of the evaluation if several dimensions are ignored can be seriously questioned. Furthermore, comparison of LVDF by the Mayo scheme ‐2003, ASE/EACVI 2009 and ASE/EACVI 2016 guidelines gave discordant results (Gottbrecht et al. 2018). Gottbrecht et al. (2018) concluded that each algorithm is seemingly effective at differentiating normal function from abnormal, and they also found ASE/EACVI 2016 guidelines to be more specific for the diagnosis of moderate or severe diastolic dysfunction relative to older algorithms. The Euro‐Filling study comparing invasively measured LV end‐diastolic pressures and noninvasively characterized LV diastolic function, reported a better agreement using the 2016 recommendations than the former, 2009 guidelines. However, the predictive value of noninvasive estimation of filling pressure was moderate, which underscores the need for further development of noninvasive characterization of LV filling pressures (Lancellotti et al. 2017).

In our inter‐rater study, the instruction to adhere to guidelines did not improve the uniformity of manual classification of diastolic function. Differences in interpretation of the guidelines regarding which algorithm to use and how to handle conflicting data probably influenced this. There was also limited agreement between manual measurements and our decision support tool. Both this and the inter‐rater discrepancy are clinically relevant since the presence or absence of elevated LV filling pressures is related to prognosis in different disease states. Although the tool is based on the same principles as advocated by the guidelines, we have introduced age‐adjusted reference limits to improve the accuracy of the classification. Several studies on normal subjects (Gentile et al. 1997; Munagala et al. 2003; Dalen et al. 2010; Caballero et al. 2015; Hagstrom et al. 2017) show that there is a substantial age dependency of E, A, e′ and E/e′, and this was also found in a study on hypertensive patients and healthy individuals (De Sutter et al. 2005). Furthermore, the limits of normal presented by recent guidelines are relatively more “tolerant” to E/A and E/e′, than to e′ alone, where a majority of elderly normal subjects may fall below the criteria of 7 cm/sec for septal and 10 cm/sec for lateral e‐velocity. We did not include tricuspid regurgitation (TR) velocity in the primary decision algorithm due to the lower feasibility of this parameter compared to left heart Doppler and tissue Doppler data (Sato et al. 2017), but TR velocity was incorporated among the parameters for the manual decision in the case of unclassified cases.

### Clinical implications

In conclusion, we present an automated decision support for evaluation of diastolic LV function by Doppler echocardiography. The algorithm is mainly based on current guidelines in that it involves multiple parameters in combination. However, the strong age dependency of several of these parameters implies that an adequate evaluation should involve age‐related normal values and cutoffs. Incorporating age dependency aspects in our program (available for use at https://liu.se/en/research/left-ventricular-diastolic-function-decision-support) facilitates the practical implementation. The large inter‐rater variation in classification in this study also underscores the usefulness of tools to support a standardized evaluation.

## Conflict of Interest

The authors have no conflicts of interest to declare.
